# Deciphering the role of SEMA4A/MAPK signaling in sepsis: insights from Mendelian randomization, transcriptomic, single-cell sequencing analyses, and vitro experiments

**DOI:** 10.3389/fcimb.2025.1606509

**Published:** 2025-07-18

**Authors:** Meng-Qin Pei, Yan-Ling Lin, Li-Ming Xu, Yu-Shen Yang, Zhen-Dong Sun, Ya-Fen Zeng, Gui-Dan Wang, He-Fan He, Li-Ying Yu

**Affiliations:** ^1^ Department of Anesthesiology, the Second Affiliated Hospital of Fujian Medical University, Quanzhou, China; ^2^ Central Laboratory, the Second Affiliated Hospital of Fujian Medical University, Quanzhou, China; ^3^ Department of Bioinformatics, School of Medical Technology and Engineering, Fujian Medical University, Fuzhou, Fujian, China

**Keywords:** SEMA4A, sepsis, Mendelian randomization, transcriptomics, single-cell sequencing

## Abstract

**Background:**

Sepsis is a condition with high mortality and multiple organ dysfunction, undergoing complex pathogenesis and limited treatment options. This study aims to uncover new therapeutic targets for sepsis

**Methods:**

Three independent transcriptomic datasets from sepsis patients in the GEO database were utilized. Batch effect correction and differential gene expression analysis were performed to identify differentially expressed genes (DEGs), followed by mendelian randomization (MR) analysis to identify sepsis-related risk genes. The intersection of DEGs and MR risk genes revealed final core sepsis genes. Gene Ontology (GO) and Kyoto Encyclopedia of Genes and Genomes (KEGG) enrichment analyses were conducted to elucidate the functional pathways of core genes. Single-cell RNA sequencing (scRNA-seq) analysis was employed to evaluate gene expression profiles across various cell types in sepsis. *In vitro* experiments were performed to validate pathways associated with the core genes.

**Results:**

We identified 307 highly expressed DEGs and 72 disease-related risk genes, culminating in the identification of three core sepsis genes including SEMA4A, LRPAP1, and NTSR1. These genes are involved in biological processes and pathways related to immune responses, such as immune rejection. scRNA-seq data indicated that three core sepsis genes are predominantly expressed in monocytes. *In vitro* experiments using THP-1 human monocytic cells validated that SEMA4A as well as the MAPK biomarker gene ERK were up-regulated in LPS-induced sepsis cells.

**Conclusion:**

This study proposes SEMA4A, LRPAP1, and NTSR1 as promising therapeutic targets for sepsis. Particularly, it underscores the crucial role of SEMA4A/MAPK in monocytes in the pathogenesis and progression of sepsis, offering valuable insights for potential treatment strategies.

## Introduction

Sepsis is a life-threatening organ dysfunction caused by a dysregulated immune response to infection, resulting in systemic inflammation, endothelial dysfunction, and multiple organ failure (Singer et al., 2016). Given its high incidence of complications and mortality (Chiu and Legrand, 2021; Modugula et al., 2025), sepsis was recognized as a global public health priority by the World Health Organization (Reinhart et al., 2017; La Via et al., 2024). Despite ongoing advancements in our understanding its pathophysiology, early diagnosis and treatment remain inadequate. Numerous studies have shown that early identification of sepsis and its associated organ dysfunction can significantly reduce severity and improve patient outcomes (Gotts and Matthay, 2016; Uffen et al., 2021). However, the significant heterogeneity in clinical manifestations often delays early diagnosis (Kim and Park, 2019). The sequential organ failure assessment (SOFA) score, a commonly used organ function assessment tool, evaluates sepsis by quantifying parameters such as respiratory rate, mental status, and systolic blood pressure (Chen et al., 2020). Notably, this scoring system demonstrates limited specificity for distinguishing infection or sepsis (Buchman et al., 2020). Furthermore, current treatments for sepsis are primarily supportive and symptomatic, relying heavily on antibiotics and vasopressor agents (Zhang and Ning, 2021). Therefore, investigating the cellular and molecular mechanisms underlying sepsis is crucial for enhancing early diagnosis and treatment strategies.

The pathogenesis of sepsis is primarily driven by immune dysregulation, which mediates disease progression (Delano and Ward, 2016). A defining feature of sepsis is the cytokine storm, which leads to excessive activation of immune cell, resulting in an imbalance in the immune response and subsequent damage or failure of multiple organ (Conway-Morris et al., 2018). Thus, exploring immune cell reprogramming in the context of sepsis can provide deeper insights into its immunopathogenesis and may uncover novel clinical treatment approaches.

Macrophages are vital components of the innate immune system and play a significant role in sepsis progression (Guo et al., 2022). Their precursors, monocytes, are derived from bone marrow and are transported through the bloodstream to various tissues and organs during infections, where they differentiate into macrophages (Epelman et al., 2014). Research indicates that macrophages typically polarize into two main types: pro-inflammatory (M1) and anti-inflammatory (M2) (Sica et al., 2015). In the early stages of sepsis, macrophage reprogramming induced by a cytokine storm shifts toward the M1 phenotype, resulting in the release of numerous inflammatory cytokines and chemokines, in which contribute to uncontrolled inflammation and organ damage (Rodriguez et al., 2019; Seman et al., 2020). Therefore, understanding the mechanisms of macrophage reprogramming in the context of immune imbalance during sepsis offers important insights for clinical intervention.

In this study, we conducted differential gene expression and mendelian randomization (MR) analyses to target three regulated risk sepsis genes (core sepsis genes: LRPAP1, NTSR1, and SEMA4A). Subsequently, single-cell sequencing analysis demonstrated high expression of these three core genes in monocytes. Moreover, we utilized immune infiltration estimation and GO/KEGG enrichment analyses to explore potential immune states and functional pathways associated with these core genes. Finally, the vitro experiments were applied to explore the most important core gene (SEMA4A) function in the sepsis. By integrating multi-omics analysis and vitro experiments, we elucidate the cellular and molecular mechanisms of SEMA4A in sepsis, laying the groundwork for novel diagnostic and therapeutic approaches.

## Methods

### Bulk RNA sequencing data acquisition and identification of differentially expressed genes

Microarray datasets from sepsis cohorts (GSE137342, GSE65682, and GSE69528) were retrieved from the Gene Expression Omnibus (GEO) database (https://www.ncbi.nlm.nih.gov/geo/). The datasets were preprocessed and merged with batch correction using R software, resulting in a final dataset comprising 390 samples from sepsis patients and 584 samples from healthy individual. Detailed characteristics of the datasets are presented in **Table 1**. Data normalization and standardization were conducted using the gene expression matrix and annotation files downloaded from the GEO database. Differential expression analysis was performed using the “limma” package to identify DEGs between the two groups, with statistical significance set at P < 0.05 and |log2 FC| > 0.5. Volcano plot and heatmap of DEGs were visualized using the “pheatmap” package. Principal component analysis (PCA) was conducted to assess the variance among the samples using the “prcomp” package in R.

**Table 1:** Dataset retrieved from the GEO database.

GEO accession	Platform	Species	Tissue	Samples (Normal control:septic shock)	Country	Type
GSE137342	GPL10558 GPL16686	*Homo sapiens*	Blood	12/22	India	RNA sequencing
GSE65682	GPL13667	*Homo sapiens*	Blood	323/479	Malta	RNA sequencing
GSE69528	GPL10558	*Homo sapiens*	Blood	55/83	USA	RNA sequencing
GSE167363	GPL24676	*Homo sapiens*	Blood	2/5	USA	Single-cell RNA sequencing

### Acquisition of exposure data and outcome data

The exposure data, expression Quantitative Trait Loci (eQTL) data, were obtained from the Genome Wide Association Study (GWAS) Catalog (https:/gwas.mrcieu.ac.uk/). Single nucleotide polymorphisms (SNPs) (p<5e-08) was utilized as instrumental variables. And linkage disequilibrium (LD) threshold of r²< 0.01 and k=10,000 kb was applied for SNP linkage disequilibrium. SNPs with an F statistic below 10 were excluded to mitigate bias from weakly associated instrumental variables. The sepsis outcome data were also acquired from the GWAS summary datasets (GWAS ID: ieu-b-4980), encompassing 11,643 sepsis cases and 474,841 controls of European ancestry (Rautanen et al., 2015). Our analysis utilized publicly available GWAS summary statistics, and as no new data collection was conducted, additional ethical approval was deemed unnecessary.

### Mendelian randomization analysis

The MR analysis was conducted using the “TwoSampleMR” software package. The primary MR approach employed was the Inverse Variance Weighting (IVW) method, hinging on three critical assumptions (Zhang et al., 2024). Firstly, the instrumental variants must exhibit a robust association with the exposure of interest. Secondly, these variants should remain unaffected confounding factors influencing both the exposure and outcome. Lastly, the impact of the variants on the outcome should operate exclusively through the exposure, without independent pathways. To evaluate potential breaches of these assumptions, we carried out multiple sensitivity analyses. Initially, both the IVW and MR-Egger methods utilized the Q-test to identify heterogeneity in associations among individual instrumental variables, aiding in the detection of potential assumption breaches. MR-Egger was further employed to assess horizontal pleiotropy using its intercept, ensuring that the genetic variants acted as independent influences on both exposure and outcome (Burgess and Thompson, 2017). Furthermore, to enhance the stability and robustness of results, additional analyses were conducted using alternative MR methods with different modeling assumptions and benefits, including the weighted median and weighted mode. In cases of inconsistent results from different MR methods, IVW is served as the primary outcome (Chen et al., 2024).

### Identification of core sepsis genes

The core sepsis genes were identified through cross-validation between disease-associated risk genes (odds ratio> 1) and DEGs. Subsequently, MR analyses were performed on all identified core sepsis genes to ascertain their causal relationships with sepsis.

### Immune cell infiltration estimation

The CIBERSORT algorithm was used to assess the differences in the infiltration levels of 22 immune cell types between sepsis patients and healthy controls (Newman et al., 2015). The “ggboxplot” R package was employed to visualize these differences. The “corrplot” R package was utilized to assess the correlation between the core sepsis genes and immune cells.

### Enrichment analysis

Enrichment analysis of core sepsis genes was conducted using the “clusterProfiler” package, including GO, KEGG, and Gene Set Enrichment Analysis (GSEA), with a filter criterion set at Pvalue < 0.05. Through GO and KEGG analyses, we aimed to elucidate the potential pathogenic mechanisms and functional pathways of core sepsis. Additionally, GSEA was performed to evaluate the activity levels of associated functions and pathways in the gene expression group. The “ggplot2” and “GOplot” packages were used to visualize the results of enrichment analysis.

### Single-cell sequencing analysis

Single-cell RNA sequencing (scRNA-seq) data from sepsis patients sourced from GSE167363 dataset were analyzed using the “Seurat” R package. The scRNA-seq dataset comprises samples from two healthy donors and five sepsis patients. Genes expressed in fewer than three single cells, cells expressing fewer than 1,000 genes, and cells with mitochondrial gene content exceeding 20% were identified and removed. The scRNA-seq data were normalized using the “NormalizeData” function. By the “RunPCA” function. Then, cell clustering analysis was performed using the “FindNeighbors” and “FindClusters” functions. Uniform Manifold Approximation and Projection (UMAP) was subsequently executed with the “RunUMAP” function, and cell clustering experiments were carried out using UMAP-1 and UMAP-2. Cell types were annotated using the “SingleR” R package, and referenced the Human Primary Cell Atlas as the dataset (Aran et al., 2019).

### Cell culture and intervention

The human monocytic cell line THP-1 was obtained from the Cell Bank of the Chinese Academy of Sciences (Shanghai, China) and cultured in RPMI-1640 medium supplemented with 10% fetal bovine serum at 37°C, under 5% CO_2_. To differentiate THP-1 cells into M0 macrophages, treatment with 100 ng/mL phorbol 12-myristate 13-acetate (PMA; Sigma, USA) was carried out. Subsequently, stimulation with lipopolysaccharide (LPS) at a concentration of 1 μg/mL for 24 hours was performed to induce the polarization of M0 macrophages into M1 macrophages (Deng et al., 2024). For target gene knock down, M0 cells were transiently transfected with SEMA4A siRNA using the riboFECT™ CP reagent (RIBOBIO) for 24 hours according to the manufacturer’s instructions. The M0 macrophages were categorized into control, LPS, and SEMA4A siRNA groups, with all groups except the control group receiving stimulation with 1 μg/mL LPS. The subsequent experiments designated these three cell groups as: CON, LPS, and SEMA4A-siRNA+LPS groups.

### Transwell assay

Cells were washed with phosphate-buffered saline (PBS) and serum-starved in RPMI-1640 medium without FBS for 12 hours. The cell migration assay was performed using a Transwell system (Costar, Corning, NY, USA) with 8-μm pore polycarbonate membranes. The lower chamber was filled with complete RPMI-1640 medium supplemented with 10% FBS as a chemoattractant. Cells were seeded onto the upper chamber at a density of 1 × 10^5^ cells in 200 µL of serum-free RPMI-1640 medium. After incubating for 24 hours at 37°C, non-migrated cells on the upper surface of the membrane were gently removed using a cotton swab. Migrated cells on the lower surface were fixed with 4% paraformaldehyde for 15 minutes, followed by staining with 0.1% crystal violet for 30 minutes. The stained cells were observed and counted under a light microscope.

### Quantitative real-time (q)PCR

Total RNA was extracted from cell samples using the RNAprep Pure Micro Kit (TIANGEN, China), followed by cDNA synthesis with the PrimeScript™ RT Reagent Kit (Takara, Japan). The expression levels of target genes were assessed using TB Green^®^ Premix Ex Taq™ II FAST qPCR (Takara, Japan) and the StepOnePlus real-time fluorescence quantitative PCR system (ABI, USA). The relative expression levels of target genes were normalized to GAPDH and calculated using the 2-ΔΔCt method. The primer sequences were shown as **Supplementary Table S1**.

### Western blotting

Equal amounts of proteins, quantified using the BCA reagent, were separated on a 10% SDS-PAGE gel and subsequently transferred to a PVDF membrane (Millipore, USA). The membranes were then blocked with 5% skimmed milk at room temperature for 2 hours and then incubated overnight at 4°C with primary antibodies, including anti-GAPDH antibody (Abcam, ab22555,1:10000), anti-SEMA4A antibody (Zenbio, 370253, 1:500), and anti-p-ERK antibody (Zenbio, 343830, 1:500). Following primary antibody incubation, the membranes were incubated with a horseradish peroxidase (HRP)-conjugated secondary antibody at room temperature for 1 hour. Immunoreactive bands were visualized using ChemiDoc imaging system (Bio-Rad).

### Statistical analysis

Statistical analysis was conducted using GraphPad Prism (version 10.0; GraphPad Software, Inc., San Diego, CA, USA). Data are presented as the mean ± standard error of the mean (SEM). Unpaired t-tests were employed to evaluate differences between the two groups. Differences among three or more groups were analyzed using one-way or two-way ANOVA, followed by Tukey’s *post-hoc* test for multiple comparisons. A p-value of less than 0.05 was considered statistically significant.

## Results

### Differential expressed genes in the sepsis

**Figure 1:**
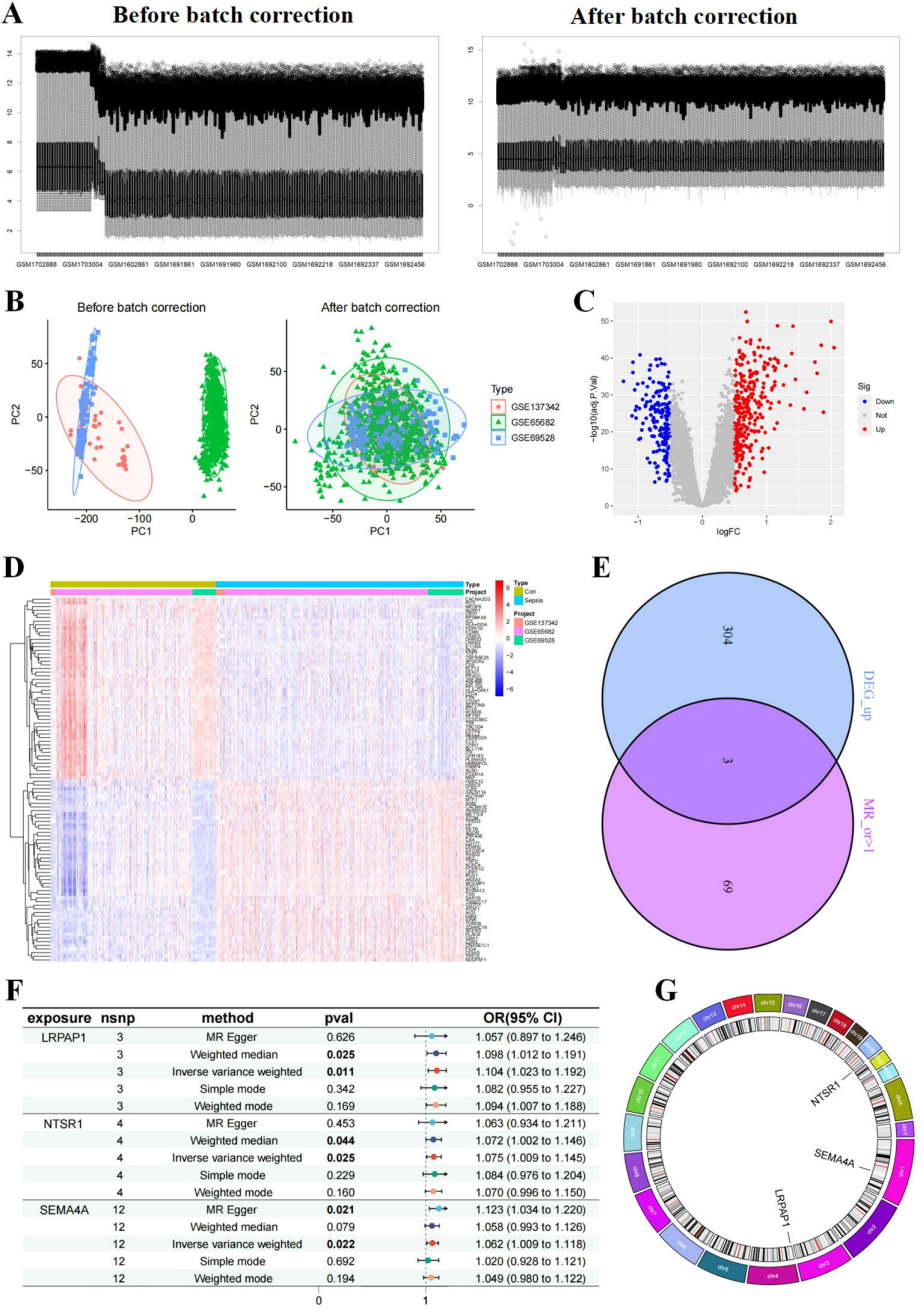
Differentially expressed genes (DEG)s identification and Mendelian randomization (MR) analyses. **(A, B)** Samples before and after batch effect removal in the merged dataset (GSE137342, GSE65682, and GSE69528). Volcano plot **(C)** and heatmap **(D)** of DEGs. **(E)** Venn diagram of up-regulated DEGs intersecting with risk genes obtained from MR analysis with OR>1, resulting in three core sepsis genes (SEMA4A, LRPAP1, and NTSR1). **(F)** MR forest and **(G)** circos plot of core sepsis genes. plot of CEGs.

Differential expression analysis between samples from sepsis patients and healthy individuals revealed genes associated with sepsis. After batch correction of three datasets (**Figure 1A**), PCA analysis indicated a distinct separation between the two groups, suggesting markedly different gene regulation patterns (**Figure 1B**). And a total of 307 up-regulated and 179 down-regulated DEGs were identified in the sepsis group compared to the control group (**Figures 1C, D**).

### Genes associated with sepsis by MR analysis

To further identify core DEGs, we conducted MR analysis to pinpoint sepsis risk genes. Utilizing published summary statistics of GWAS data, we identified 72 genes associated with sepsis through two-sample MR analysis. Notably, the analysis highlighted LRPAP1, NTSR1, and SEMA4A as up-regulated risk genes in sepsis (core sepsis genes, **Figure 1E**). Subsequent MR analysis on these three core sepsis genes a significant positive relationship with sepsis for all three genes on sepsis based on the IVW method: LRPAP1 (OR=1.104; 95% CI: [1.023~1.192]; P = 0.011), NTSR1 (OR=1.075; 95% CI: [1.009~1.145]; P = 0.025), and SEMA4A (OR=1.062; 95% CI: [1.009~1.118]; P = 0.022) (**Figure 1F**). Additional validation using MR-Egger, simple model, weighted median, and weighted model methods confirmed the increased sepsis risk associated with these three core genes (OR > 1, Pvalue < 0.05). Tests for heterogeneity and pleiotropy indicated no significant impact (P > 0.05) on the three genes. Chromosomal distribution showed that LRPAP1, NTSR1, and SEMA4A were located on different chromosomes (4, 20, and 1, respectively) (**Figure 1G**).

### Immune cell infiltration changes in sepsis

**Figure 2:**
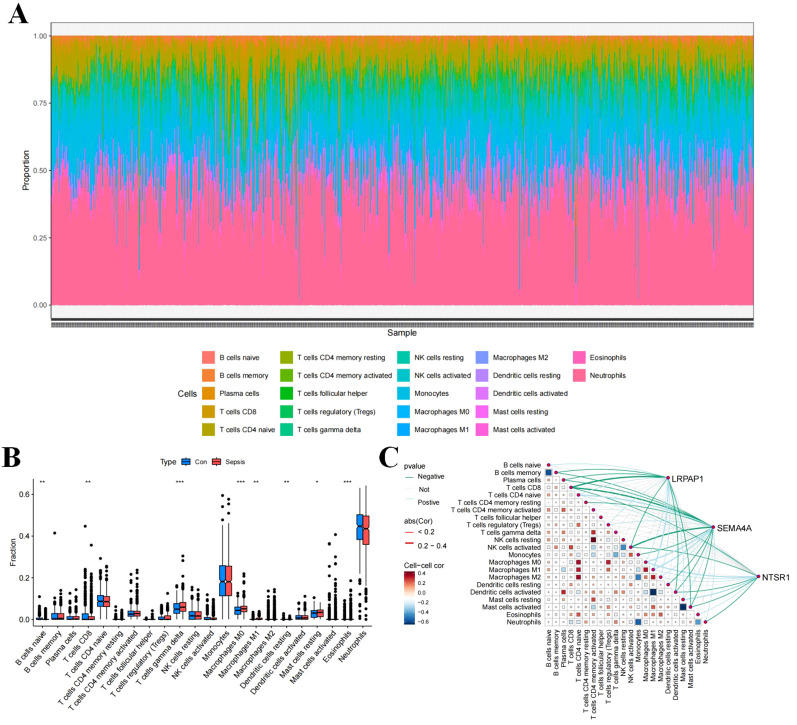
Immune cell infiltration in sepsis. **(A)** Stacked histogram of the proportions of immune cells between the sepsis group and the control group. **(B)** Box plot showing the comparison of 22 types of immune cells between the sepsis group and the control group. **(C)** Heatmap showing the correlation between 22 types of immune cells and the three core sepsis genes (SEMA4A, LRPAP1, and NTSR1).

To evaluate the immune cell composition within the septic microenvironment, the CIBERSORT algorithm was employed to assess the percentages of 22 immune cell types in each sample (**Figure 2A**). Statistical analysis showed significant differences in various immune cell subtypes between sepsis and control samples, including B cells naive, T cells CD8, T cells gamma delta, Macrophages M0, Macrophages M1, Dendritic cells resting, Mast cells resting, and Eosinophils, between sepsis and control samples (**Figure 2B**). Correlation analysis indicated positive correlations of LRPAP1, NTSR1, and SEMA4A with T cells CD4 naive, Macrophages M0, Macrophages M1, Macrophages M2, and Mast cells resting, while negative correlations were observed with T cells CD8, NK cells activated, Dendritic cells activated, and Mast cells activated (**Figure 2C**).

### Enrichment analysis of core sepsis genes

**Figure 3:**
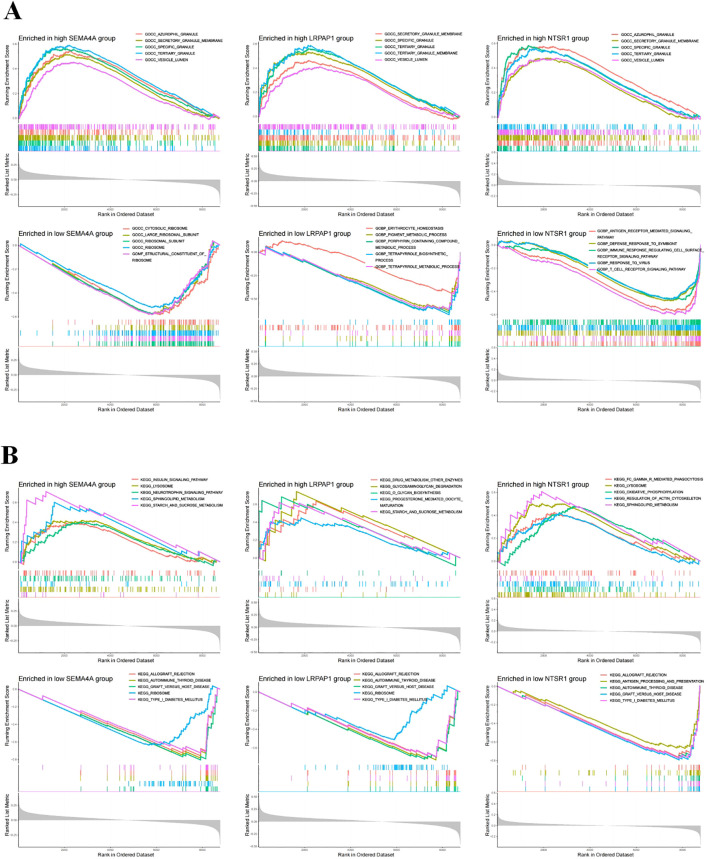
Functional enrichment analysis. **(A)** The top five active biological functions in core sepsis genes high or low expression group. **(B)** The top 5 active pathways in core sepsis genes high or low expression group.

To elucidate potential mechanisms associated with three core genes, we conducted GSEA to assess GO and KEGG functional enrichment. In the high expression group of SEMA4A, top five active GO biological functions included zurophil granule, secretory granule membrane, specific granule, tertiary granule, and vesicle lumen (**Figure 3**). The top active KEGG pathways in the high expression group of SEMA4A comprised the insulin signaling pathway, lysosome, neurotrophin signaling pathway, sphingolipid metabolism, and starch and sucrose metabolism. While the low expression group of SEMA4A exhibited active GO biological functions and pathways such as cytosolic ribosome, large ribosomal subunit, ribosomal subunit, ribosome, and structural constituent of ribosome, along with KEGG pathways like allograft rejection, autoimmune thyroid disease, graft versus host disease, ribosome, and type I diabetes mellitus. The high or low expression groups of LRPAP1 and NTSR1 displayed different regulatory pathways (**Figure 3**).

### Validation group differential analysis

**Figure 4:**
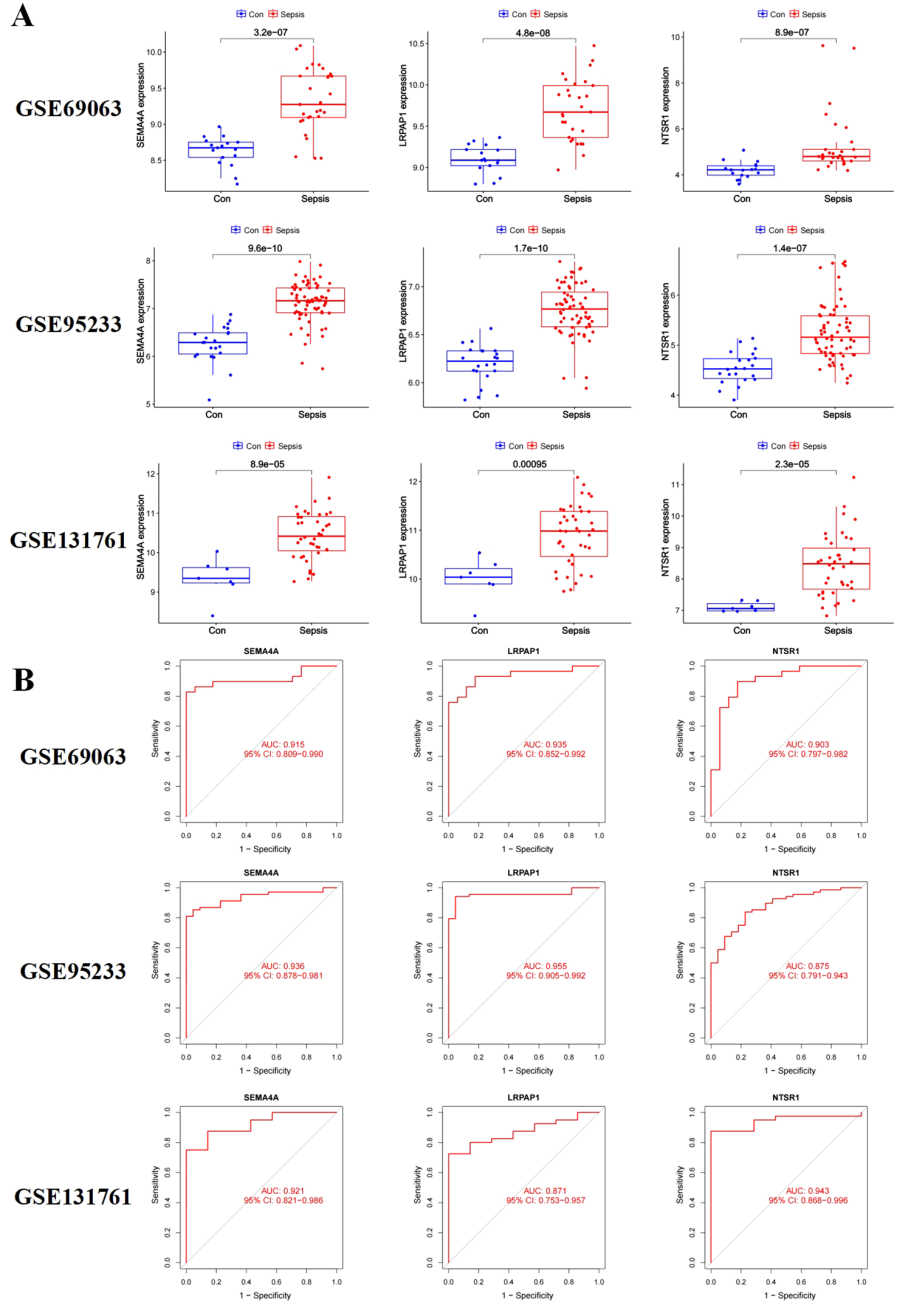
Validation of the three core sepsis genes (SEMA4A, LRPAP1, and NTSR1) in additional transcriptomic datasets. **(A)** Gene expression in three validation datasets (GSE69063, GSE95233, and GSE131761). **(B)** ROC analysis of core sepsis genes in three validation datasets.

To validate the expression of the three core sepsis genes, we utilized three additional GEO datasets (GSE69063, GSE95233, and GSE131761) for differential expression analysis. The results confirmed significantly higher expression levels of LRPAP1, NTSR1, and SEMA4A in these additional sepsis samples compared to the control group (**Figure 4A**). Moreover, the area under the ROC curve (AUC) demonstrated high reliability for all three genes (**Figure 4B**).

### Single-cell RNA sequencing analysis

**Figure 5:**
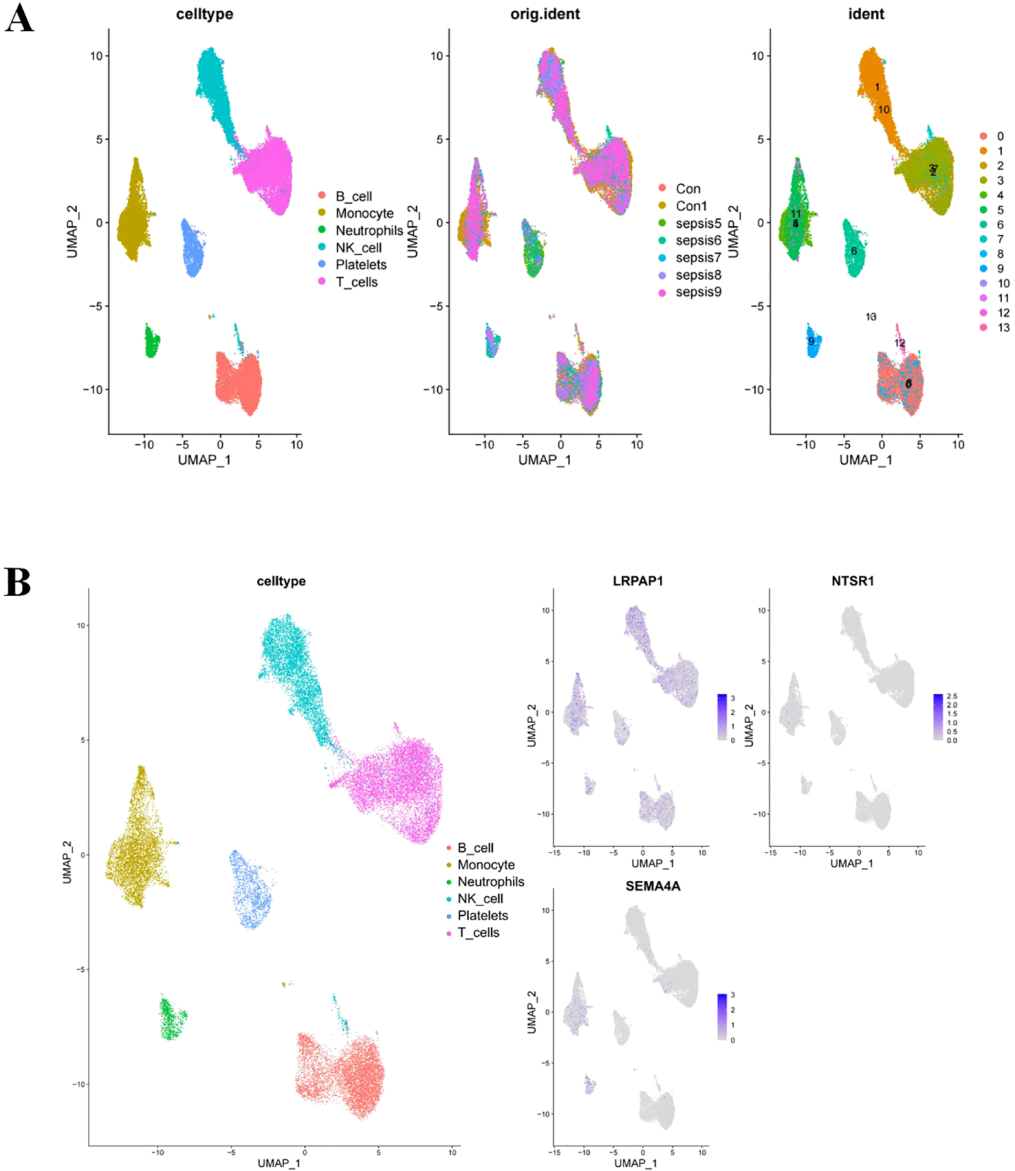
Core sepsis gene (SEMA4A, LRPAP1, and NTSR1) expression in various cell types using Single-cell RNA sequencing analysis. **(A)** Visualization of cell clustering in sepsis using UMAP. **(B)** Distribution of core sepsis genes in cell clusters.

ScRNA-seq was employed to characterize gene expression profiles in cell types of sepsis. Analysis revealed six distinct cell types including B cells, monocytes, neutrophils, natural killer cells, platelets cells and T cells across samples from healthy donors and sepsis (**Figure 5A**). LRPAP1 exhibited high expression across all cell types, while SEMA4A showed predominant expression in monocytes and neutrophils, and NTSR1 was primarily found in monocytes (**Figure 5B**). The common high expression of the three core sepsis genes in monocytes underscores the significant role of monocytes in the progression of sepsis.

### Verification of core sepsis gene expression

**Figure 6:**
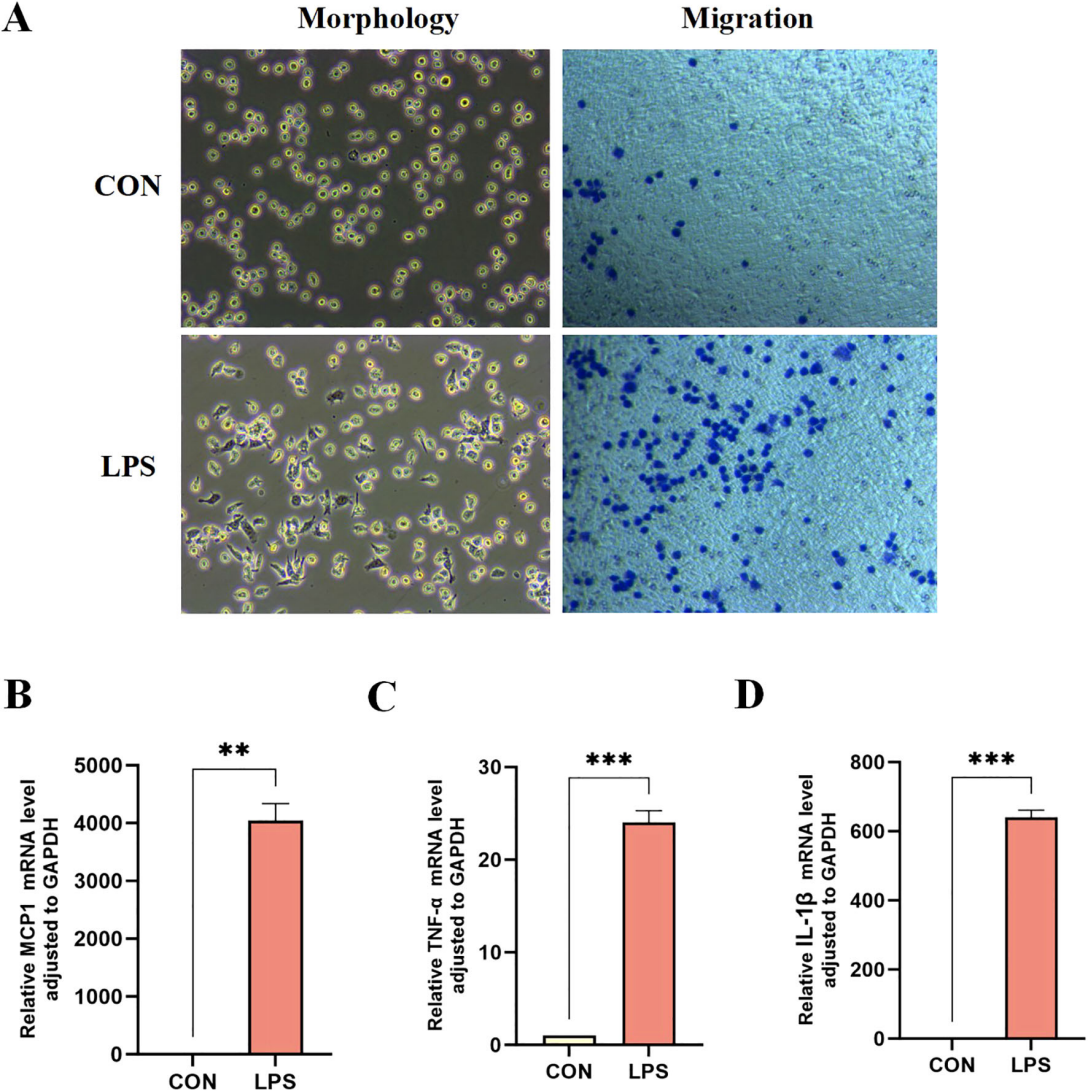
Differentiation of THP-1 monocytes into M1 macrophages. **(A)** Morphological and migration experiments of THP1 cells in two groups. mRNA expression levels of pro-inflammatory factors MCP-1 **(B)**, TNF-α **(C)**, and IL-1β **(D)** in the two groups of THP-1 cells. **P < 0.01, ***P < 0.001.

*In vitro* experiments using LPS-stimulated THP-1 human monocytic leukemia cells were conducted to verify the expression of core sepsis genes. LPS-treated THP-1 cells exhibited morphological characteristics typical of M1 macrophages, including cellular polymorphism, abundant pseudopodia, distinct branching, and characteristically enhanced migratory capacity (**Figure 6A**). The elevated expression of pro-inflammatory factors further substantiated the successful differentiation of THP-1 monocytes into M1 macrophages (**Figures 6B-D**).

**Figure 7:**
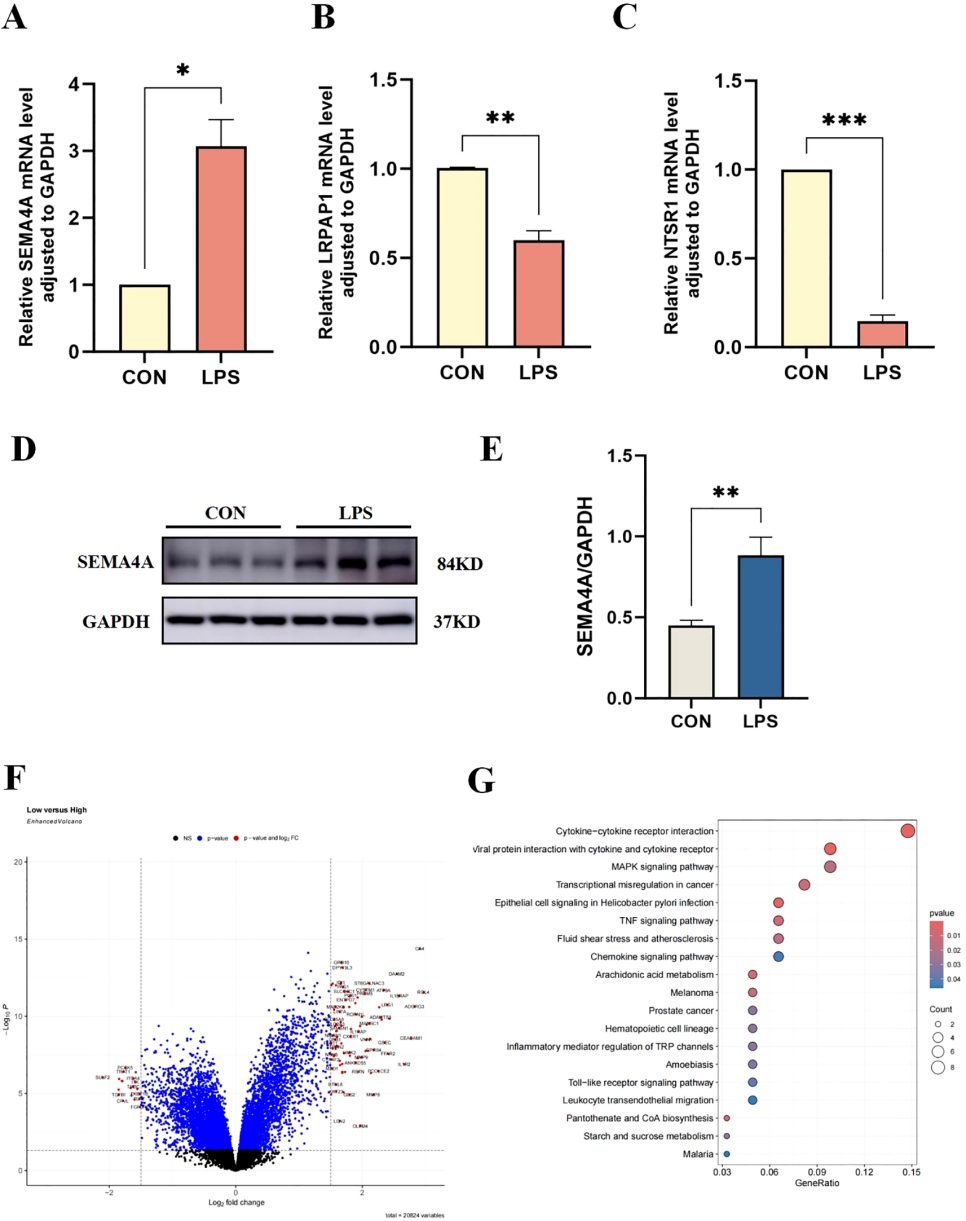
Verification of core sepsis gene (SEMA4A, LRPAP1, and NTSR1) expression *in vitro* model. **(A-C)** mRNA expression levels of CEGs in the control and LPS-induced groups of THP-1 cells. **(D, E)** Protein expression levels of SEMA4A in the two groups of THP-1 cells. Volcano plot **(F)** and pathway enrichment **(G)** of differentially expressed genes between groups of high- and low-SEMA4A expression. *P < 0.05, **P < 0.01, ***P < 0.001.

Among three core sepsis genes, only SEMA4A was upregulated in the model group (**Figures 7A, D, E**), whereas LRPAP1 and NTSR1 exhibited decreased expressions (**Figures 7B, C**), contrary to previous results. Thus, further investigation was directed towards SEMA4A.

### Validation of the roles of SEMA4A/MAPK signaling pathway in the progression of monocytes during sepsis

Dataset samples were categorized into high- and low-SEMA4A expression groups based on the median expression of SEMA4. Subsequent differential expression and enrichment analyses revealed a significant enrichment in the MAPK signaling pathway (**Figures 7F, G**), suggesting that SEMA4A may influence sepsis progression through the MAPK signaling pathway.

**Figure 8:**
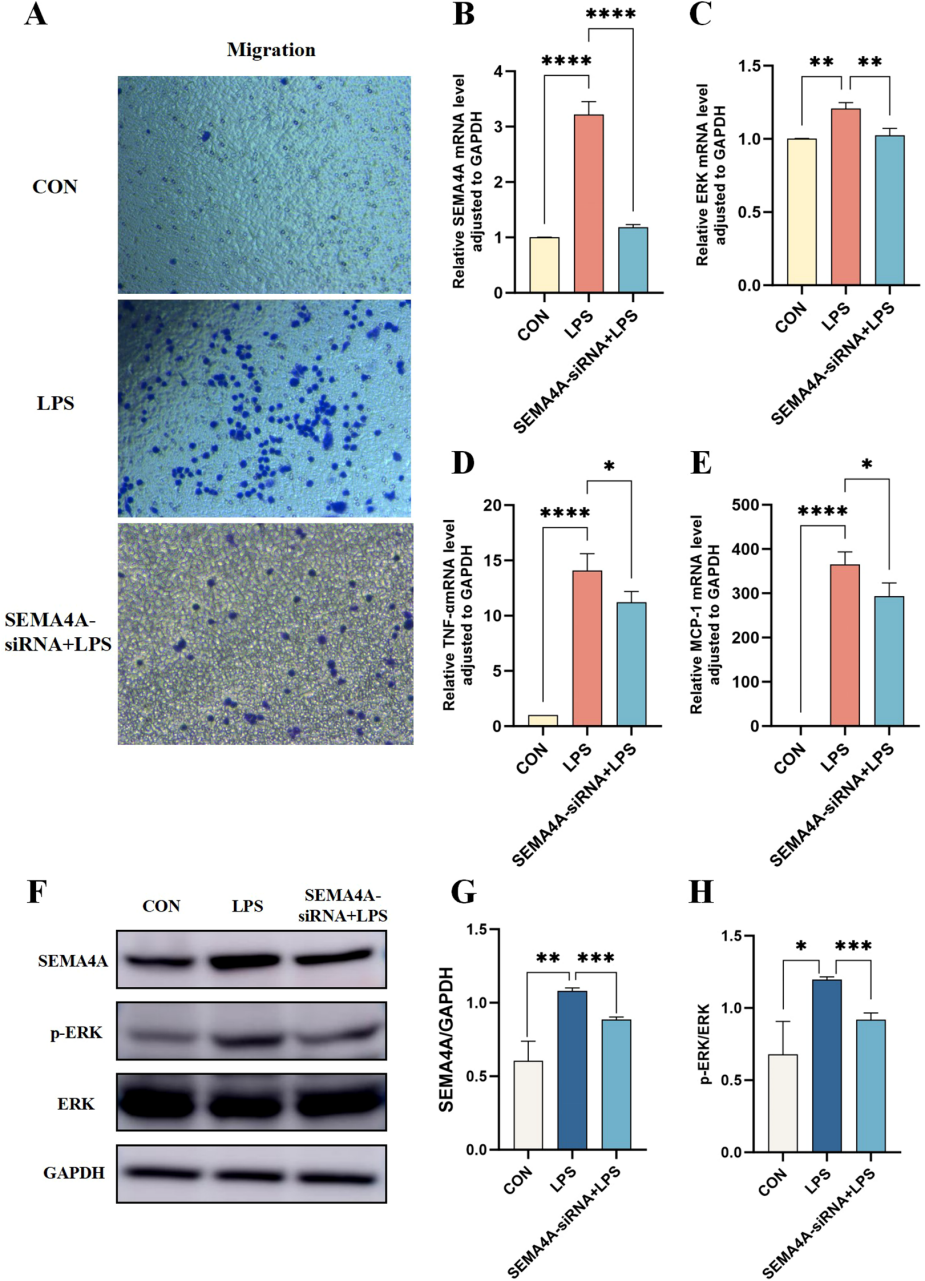
Validation of the roles of SEMA4A/MAPK signaling pathway in the progression of monocytes during sepsis *in vitro* model. **(A)** Migration experiments of THP1 cells in the control, LPS-induced and SEMA4A-siRNA+LPS treated groups of THP-1 cells. mRNA expression levels of SEMA4A **(B)**, ERK **(C)**, TNF-α **(D)** and MCP-1 **(E)** in the three groups of THP-1 cells. **(F-H)** Protein expression levels of SEMA4A, p-ERK and ERK in the three groups of THP-1 cells. *P < 0.05, **P < 0.01, ***P < 0.001, ****P < 0.0001.

To further elucidate the involvement of the SEMA4A/MAPK pathway in sepsis, we developed a THP-1 cell model with silenced SEMA4A expression. The results confirmed a decreased in SEMA4A expression at both mRNA and protein levels compared to the LPS group (**Figures 8B, F**). In septic conditions, the migratory capacity of M1-type macrophages was significantly attenuated following SEMA4A silencing (**Figure 8A**). Notably, the representative biomarker of the classical MAPK pathway, ERK, exhibited a substantial increase mRNA expression in the LPS group, which subsequently decreased following SEMA4A silencing (**Figure 8C**). A parallel trend was observed in the phosphorylation status of ERK protein (**Figure 8E**), while total ERK protein levels remained unaltered (**Figures 8F–H**). Moreover, the reduction in inflammation following SEMA4A silencing was evident (**Figures 8D, E**). These findings indicate the crucial role of the SEMA4A/MAPK signaling pathway in the modulation of monocyte polarization during sepsis.

## Discussion

Sepsis, one of the leading global causes of mortality (Rudd et al., 2020), poses a significant challenge in clinical management due to its complex pathogenesis. This study aims to provide new insights into sepsis mechanisms by integrating transcriptomics, single-cell sequencing, MR analysis, and vitro experiments, paving the way for novel therapeutic avenues.

Three core genes were identified associated with sepsis risk. NTSR1 is situated on chromosome 20 (20q13) and encodes a protein with 424 amino acids (aa) in rats and 418 aa in humans and mice (Kyriatzis et al., 2024). It acts as a primary receptor for neurotensin (NT), exerting effects in both the central and peripheral nervous systems, such as blood pressure reduction, analgesia, and hypothermia (Rioux et al., 1982; Roussy et al., 2008; Ramirez-Virella and Leinninger, 2021). Studies have linked NTSR1 overexpression to inflammatory bowel disease progression and neuroinflammatory responses (Gui et al., 2013; Gu et al., 2024), suggesting a potential role in inflammatory conditions like sepsis. LRPAP1, located on chromosome 4, serves as a molecular chaperone for the low-density lipoprotein receptor (LDLR) (Liu et al., 2022). It interacts with various surface lipoprotein receptors involved in cholesterol-lipoprotein complex internalization, indicating its role as a universal antagonist and chaperone for lipoprotein receptors (Carter, 2007). Given the critical role of alterations in lipid metabolism in sepsis pathophysiology (Amunugama et al., 2021), our study sheds light on the regulatory function of LRPAP1 in lipid metabolism during sepsis. SEMA4A, located on chromosome 1, belongs to the semaphoring protein (Suzuki et al., 2008). which is crucial for neuronal and immune functions. It regulates neuronal activity as an axon guidance molecule and modulates immune cell activation and function (Iyer and Chapoval, 2018). Recent attention has focused on the regulatory role of SEMA4A in immune cells, particularly in conditions like multiple sclerosis. As evidence suggested, SEMA4A is involved in the activation of helper T cells (Th) during the initiation phase and also accelerates Th17 cell-mediated neuroinflammation during the effector phase (Koda et al., 2020). While the specific role of SEMA4A in sepsis remains unclear. Our study unveils novel potential research targets for exploring their involvements in sepsis pathogenesis.

To gain deeper insights into the functions and pathways influenced by three core genes in sepsis, we conducted GO and KEGG enrichments among groups with high and low expression of NTSR1, LRPAP1, and SEMA4A genes. These analyses provide valuable insights for future research. In the high gene expression groups, primary GO terms were notably enriched in various secretory granules, including tertiary granule and vesicle lumen. Conversely, the low gene expression groups exhibited significant enrichment in pathways related to immune responses, such as allograft rejection and graft-versus-host disease. Moreover, our assessment of immune cell infiltration status in sepsis revealed substantial changes in immune cells in sepsis progression. Additionally, correlation studies between core sepsis genes and immune cells unveil potential roles and regulatory effects of NTSR1, LRPAP1, and SEMA4A on immune cells in sepsis.

The scRNA-seq analysis revealed that B cells, monocytes, neutrophils, natural killer cells, and platelets were main cell types in sepsis. Notably, NTSR1, LRPAP1, and SEMA4A exhibited high expression level in monocytes. Monocytes are heterogeneous blood cells that play a critical role in antibacterial immune defense and tissue healing (Kratofil et al., 2017). They originate in the bone marrow and migrate to infection sites, where they mature into macrophages or dendritic cells to safeguard tissues (Rimmelé et al., 2016). However, under septic immune dysregulation environment, monocytes can act as a double-edged sword, exacerbating infection and tissue damage (Maneta et al., 2023), underscoring their critical role in sepsis-associated immune dysregulation. Targeting specific pathways involving monocytes could offer a promising avenue for future research.

Subsequently, we established an *in vitro* sepsis inflammation model using THP1 cells to investigate the expression of core sepsis genes in the model group. While the differential expression analysis of the dataset indicated that upregulation of NTSR1, LRPAP1, and SEMA4A in sepsis, our *in vitro* experiments revealed that only SEMA4A was upregulated in the model group. The observed discrepancies in NTSR1 and LRPAP1 expression levels between bioinformatics predictions and experimental results may be attributed to several methodological and biological factors. Firstly, our analysis utilized datasets comprising heterogeneous cell populations, where monocyte/macrophage-specific expression profiles may be obscured by bulk sequencing approaches. This fundamental difference in analytical resolution between whole blood sequencing and single-cell profiling could account for the observed variations. Secondly, the datasets originated from different countries with significant demographic variations, which may contribute to the observed discrepancies. Finally, at the experimental level, the duration of LPS stimulation during the induction of M0 macrophages to M1 macrophages represents a critical variable influencing gene expression dynamics. The absence of multiple LPS treatment time points in our experimental design may have constrained our capacity to capture temporal regulation patterns of NTSR1 and LRPAP1 expression. Notably, SEMA4A emerged as a potentially novel target warranting further exploration in sepsis research.

To further explore the role of SEMA4A in the development and progression of sepsis, we conducted differential expression analysis and KEGG enrichment on additional datasets by categorizing samples into high- and low- SEMA4A expression groups The results demonstrated significant enrichment in the MAPK signaling pathway. MAPK, a member of the serine-threonine kinase superfamily, acts as a principal signaling pathway for cell proliferation, extending from the cell membrane to the nucleus. The MAPK family encompasses three primary subfamilies: extracellular signal-regulated kinase (ERK MAPK, Ras/Raf1/MEK/ERK), c-Jun N-terminal or stress-activated protein kinase (JNK or SAPK), and MAPK14 (Fang and Richardson, 2005). Moreover, the MAPK signaling pathway is a classic inflammatory response pathway and is implicated in the initiation and progression of sepsis (Liu et al., 2024). Our subsequent transfection experiments, silencing SEMA4A expression, further confirmed that SEMA4A can regulate the MAPK signaling pathway, positioning it as a novel upstream regulatory molecule in the pathological context of sepsis.

However, we must acknowledge that this study has some limitations. Firstly, the exclusion of genes not intersecting with disease-related genes obtained from MR analysis among the DEGs may result in oversight of key molecules pertinent to sepsis pathology. Secondly, the experimental validation of core sepsis genes was restricted to THP1 cells, necessitating further validation at the animal level.

## Conclusion

This study offers a comprehensive analysis of genes contributing to sepsis by combining transcriptomics, MR, single-cell sequencing, and *in vitro* experiments, with a particular emphasis on SEMA4A, LRPAP1, and NTSR1. Notably, the SEMA4A/MAPK signaling pathway is highlighted as pivotal in driving the initiation and progression of sepsis within monocytes. This finding provides new insights into the understanding of sepsis pathogenesis and potential treatment strategies.

## Data Availability

The data supporting this study are available in [NCBI Gene Expression Omnibus] under accession numbers [e.g., GSE137342, GSE65682, GSE69528, and GSE167363]. These datasets were derived from existing public resources and do not require new deposition.
