# Supplementary material for: Deciphering the role of SEMA4A/MAPK signaling in sepsis: insights from Mendelian randomization, transcriptomic, single-cell sequencing analyses, and vitro experiments
Source: Front Cell Infect Microbiol. 2025 Jul 18;15:1606509. doi: 10.3389/fcimb.2025.1606509 (PMC12313560; doi:10.3389/fcimb.2025.1606509)
Supplement: Supplementary file 1 [file Table1.docx]

**Supplementary Table 1 Primers in quantitative real-time PCR**

| Gene | Forward primer (5′-3′) | Reverse primer (5′-3′) |
| --- | --- | --- |
| SEMA4A | TTGGTGGATGGGATGCTCTATTCTG | GGAGGAAGTTGTCGGTCTTGAGG |
| NTSR1 | CCGTCAAGGTCGTCATACAGGTC | TGGCGATGATGGTGTTCAGGAC |
| LRPAP1 | GCTACTGCTGCTGCTCTTCCTC | GGCTTGGGCTGGTTCTTCTCC |
| ERK | ACCGTGACCTCAAGCCTTCC | ATCATGGTCTGGATCTGCAACAC |
| TNF-α | GCTGCACTTTGGAGTGATCG | GGGTTTGCTACAACATGGGC |
| IL-1β | CCACAGACCTTCCAGGAGAA | GTGATCGTACAGGTGCATCG |
| IL-6 | GGAGACTTGCCTGGTGAAAA | GTCAGGGGTGGTTATTGCAT |
| MCP1 | CAGCCAGATGCAATCAATGCC | TGGAATCCTGAACCCACTTCT |
| GAPDH | GGACTCATGACCACAGTCCAT | CAGGGATGATGTTCTGGAGAG |
